# BCAP Is an Interferon-Stimulated Gene That Enhances Type I Interferon Activity in Response to Lipopolysaccharide

**DOI:** 10.3390/ijms26157034

**Published:** 2025-07-22

**Authors:** Marianna Di Rosa, Giulia Maria Piperno, Alessandra Tesser, Alessia Pin, Giada Sospiro, Erica Valencic, Valentina Boz, Serena Pastore, Alberto Tommasini, Federica Benvenuti

**Affiliations:** 1 Department of Medical Sciences, University of Trieste, 34129 Trieste, Italy; 2 Cellular Immunology, International Centre for Genetic Engineering and Biotechnology (ICGEB), 34149 Trieste, Italy; 3 Institute for Maternal and Child Health IRCCS Burlo Garofolo, 34137 Trieste, Italy

**Keywords:** type I interferon, interferon-stimulated genes (ISG), B-cell adapter for PI3K (BCAP), lipopolysaccharide (LPS), systemic lupus erythematosus (SLE)

## Abstract

The B-cell adapter for PI3K (BCAP) is a protein that connects membrane receptor signaling to the PI3K pathway. In fibroblasts or dendritic cells, priming the cGAS nucleic-acid-sensing pathway increases BCAP expression and enhances type I interferon (IFN-I) production upon lipopolysaccharide (LPS) stimulation. These findings corroborate the idea that BCAP may bias cytokine production toward IFN during inflammation, indicating its potential involvement in IFN-driven diseases like systemic lupus erythematosus (SLE). We investigate the role of BCAP in regulating the inflammatory response in SLE and its relationship with IFN-mediated inflammation. BCAP gene expression and IFN signature were analyzed in 36 subjects with SLE and 20 healthy controls. Two cellular models were used to assess BCAP’s role in LPS response and IFN signaling after cGAS stimulation. We found a correlation between BCAP and interferon-stimulated gene (ISG) expression in SLE. In a cellular model, tofacitinib and anifrolumab, acting as IFN signaling “inhibitors”, blocked BCAP overexpression triggered by cGAS, confirming BCAP as an ISG. Additional studies in BCAP^−/−^ cells revealed that, in the absence of BCAP, these cells exhibited diminished IFN production upon LPS stimulation following prior exposure to cGAMP. Overall, BCAP is an ISG that acts as a positive regulator of Toll-like receptor 4-mediated IFN production. We speculate that its increased expression in SLE may contribute to a positive feedback loop, enhancing IFN production during bacterial infections.

## 1. Introduction

The B-cell adapter for PI3K (BCAP) is a protein connecting various membrane stimuli with PI3K signaling. Further, it presents a functional N-terminal Toll-IL1-receptor (TIR) homology domain that can associate with MyD88 and TIR adaptor protein (TIRAP) linking Toll-like receptor (TLR) signaling to PI3K activation and regulating the inflammatory response [1,2]. According to recent data, BCAP deficiency results in reduced PI3K activation, reduced expression of Ifit1 and Isg15, and reduced production of type I interferon (IFN-I) after Toll-like receptor 9 (TLR9) stimulation [3,4]. Conversely, we previously indicated that background activation of the cGAS-STING-TBK1 pathway is associated with elevated BCAP expression and augmented IFN-I production following lipopolysaccharide (LPS) stimulation. This behavior was observed in both DNase2-deficient fibroblasts, which exhibit constant activation of the cGAS-STING-TBK1 pathway due to a genetic defect, and HoxB8 cells, which were primed under experimental conditions. These results support a possible role of BCAP in connecting the LPS-Toll-like receptor 4 (TLR4) pathway to the cGAS-STING-TBK1 cytoplasmic nucleic-acid-response pathway [5]. They also indicate that BCAP activation may reroute cellular responses toward IFN-I production rather than inflammatory cytokines such as interleukin 1 (IL-1), interleukin 6 (IL-6), and tumor necrosis factor α (TNFα). Nevertheless, although a correlation between BCAP expression and heightened IFN response has been shown, the nature of the interaction between these two occurrences remains unclear.

In order to ascertain whether an increase in BCAP is associated with unregulated production of IFN in vivo, we examined the correlation between IFN pathway activation and BCAP gene (*PIK3AP1*) expression in peripheral blood cells from pediatric patients with SLE and healthy controls. Building the experimental design based on previous experience with DNase2-deficient fibroblasts, we also stimulated healthy controls’ fibroblasts in vitro to study the relationship between the expression of IFN and BCAP.

In addition, we generated BCAP^−/−^ murine HoxB8-derived macrophages and assessed their production of IFN following LPS stimulation to determine the role of BCAP in regulating the inflammatory response. Our findings provide additional pieces of evidence for the idea that *PIK3AP1* acts as an IFN-stimulated gene (ISG) that can regulate a positive feedback loop, leading to increased production of IFN in response to bacterial LPS stimulation.

## 2. Results

### 2.1. PIK3AP1 Expression and IFN Score Values Are Higher in Patients with SLE than in Controls

**Figure 1:**
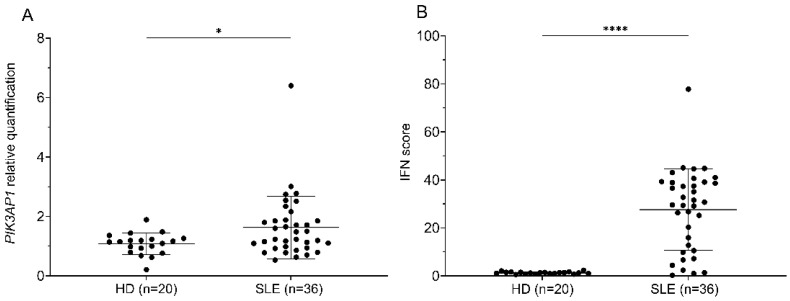
Representation of *PIK3AP1* gene expression levels (**A**) and IFN score (**B**) in controls (HD) and patients (SLE), as calculated by qPCR. On the x-axis are shown the following two groups: controls (HD, n = 20) and patients (SLE, n = 36). Each dot represents a single value calculated for the HD and SLE groups. The results are reported as the mean ± SD. On the y-axis are represented the *PIK3AP1* gene expression levels (**A**) and the IFN score values (**B**). Mann–Whitney test *p*-values are shown (* *p* < 0.05 and **** *p* < 0.0001).

To establish a link between the expression of BCAP and that of type I IFN-stimulated genes in human samples, we measured *PIK3AP1* expression in our cohort of subjects with SLE and correlated it with the IFN score (based on the expression of *IFI27*, *IFI44L*, *IFIT1*, *ISG15*, *RSAD2*, and *SIGLEC1*). The values obtained from whole-blood RNA are represented graphically by boxplots. A Mann–Whitney test showed a significant difference between the group of patients with SLE and controls, both in terms of *PIK3AP1* gene expression levels (*p*-value = 0.04; mean “HD” = 1.1, and mean “SLE” = 1.6) and IFN score (*p*-value < 0.0001; mean “HD” = 1.3, and mean “SLE” = 26.3) (Figure 1A,B).

**Figure 2:**
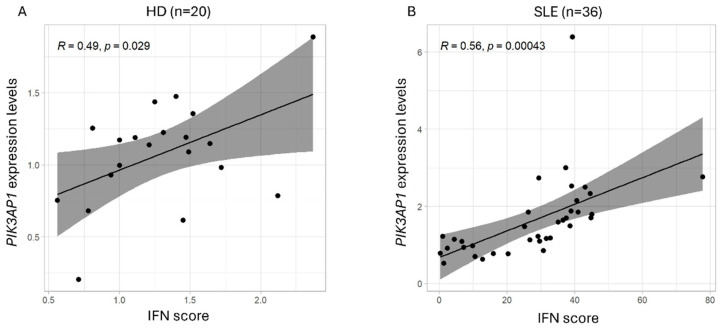
Graphical representation of the correlation between *PIK3AP1* expression and IFN score values in (**A**) control (HD, n = 20) and (**B**) patient (SLE, n = 36) groups, as calculated by qPCR. On the x-axis are shown the IFN score values and, on the y-axis, the *PIK3AP1* gene expression levels. “R” indicates the correlation coefficient and “*p*” the statistical significance calculated with Pearson’s test.

Pearson’s test showed a moderate direct correlation between *PIK3AP1* expression values and IFN, which tended to be stronger in patients with SLE than in controls. Nevertheless, the confidence interval (CI) is elevated for both groups, indicating large variation across the samples (HD: R = 0.49, *p*-value = 0.029, and CI = 0.05987417 ± 0.76603819; SLE: R = 0.56, *p*-value = 0.00043, and CI = 0.2786213 ± 0.7480702) (Figure 2).

### 2.2. PIK3AP1 Is an ISG, and Its Hyperexpression Following cGAS Priming Is Inhibited by IFN Signaling Blockade via Tofacitinib or Anifrolumab

**Figure 3:**
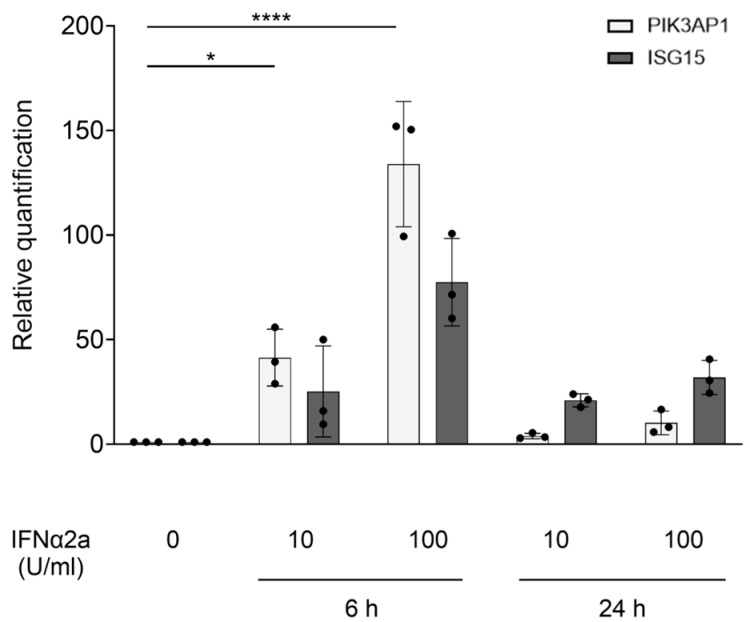
Graphical representation of the results obtained for fibroblasts from controls following stimulation with IFNα2a (10, 100 U/mL). On the x-axis are shown the experimental conditions (IFNα2a concentrations and different time points) and, on the y-axis, the *PIK3AP1* and *ISG15* gene expression levels. The results are reported as the mean ± SD (n = 3 independent experiments). One-way ANOVA test (with multiple comparison corrections) *p*-values are shown (* *p* < 0.05 and **** *p* < 0.0001).

To delve into the mechanism that connects elevated IFN-I signaling to BCAP expression, we analyzed cultured fibroblasts derived from healthy controls. Fibroblasts were stimulated with increasing concentrations of IFNα2a. The results show that 6 h after the stimulation, there was a significant rise in *PIK3AP1* expression (*p*-value = 0.0207 and *p*-value < 0.0001, respectively, for 10 U/mL and 100 U/mL of IFNα2a), which thereafter declined and returned to near basal levels within 24 h, and for an established ISG, *ISG15* (Figure 3). This kinetic profile supports the hypothesis that *PIK3AP1* may perform its function as an ISG during the early phase of the inflammatory process.

**Figure 4:**
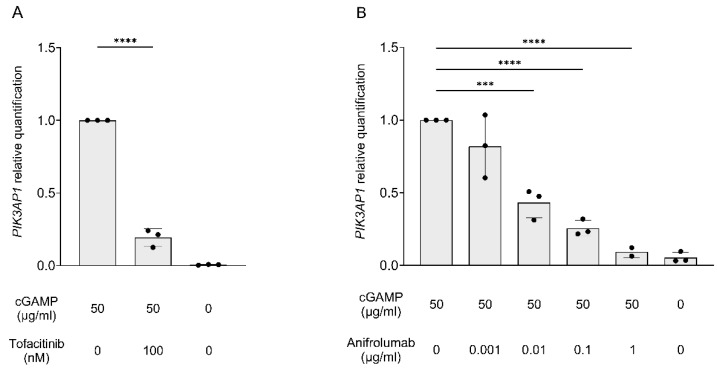
Graphical representation of the results obtained for fibroblasts from controls following treatment with cGAMP (50 μg/mL) in combination with (**A**) tofacitinib (100 nM) or (**B**) anifrolumab (0.001, 0.01, 0.1, 1 μg/mL). On the x-axis are shown the experimental conditions (cGAMP, tofacitinib, and anifrolumab concentrations) and on the y-axis the *PIK3AP1* gene expression levels. The results are reported as the mean ± SD (n = 3 independent experiments). One-way ANOVA test (with multiple comparison corrections) *p*-values are shown (*** *p* < 0.001 and **** *p* < 0.0001).

We reproduced the experiment by stimulating cells with cGAMP to activate cGAS-STING signaling and induce IFN synthesis, instead of using IFN directly. We observed that stimulation with cGAMP also induced an upregulation of *PIK3AP1* expression. To confirm that also in this setting BCAP upregulation depends on the effects of IFN-I, we treated cells with either the JAK inhibitor tofacitinib, which blocks IFN signaling downstream of the IFN-I receptor (IFNAR), or the monoclonal antibody anifrolumab, which prevents the binding of IFN-I to IFNAR. Both treatments strongly reduced *PI3KAP1* expression after stimulation with cGAMP (Figure 4A). Furthermore, the impact of IFN-I receptor blockade by anifrolumab was dose-dependent, leading to an almost complete shutdown of *PIK3AP1* at the highest doses (Figure 4B). These findings indicate that *PIK3AP1* behaves as an ISG.

### 2.3. BCAP Skews LPS-Stimulated Cells Toward IFN-I Production

**Figure 5:**
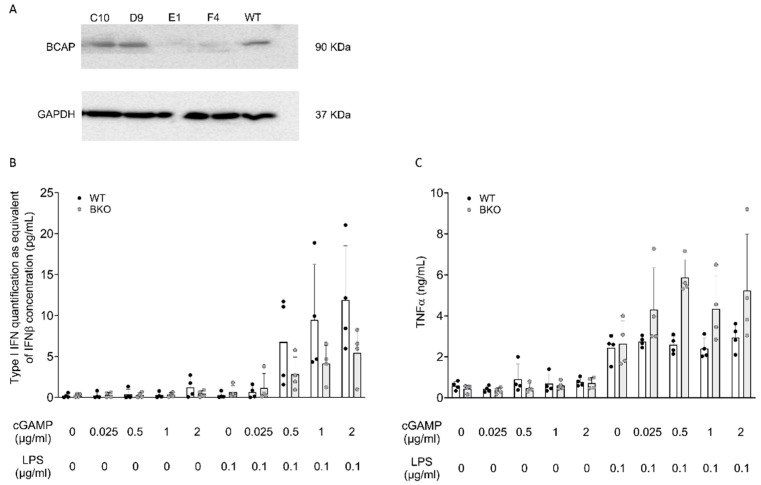
(**A**) BCAP protein expression in HoxB8-transduced cells after differentiation in macrophages. The blot shows a representative image of single clones after BCAP infection compared with the WT as a control. The original blot is reported in Figure S1. (**B**,**C**) 2 × 10^5^ cells (WT: wild type; BKO: BCAP^−/−^ was primed with graded doses of cGAMP (0.025, 0.5, 1, 2 μg/mL) for 24 h, washed, and, subsequently, stimulated with LPS (0.1 μg/mL) in fresh medium for a further 24 h. The results of both BKO clones (E1 and F4) are shown together. Secreted IFN-I was evaluated by a B16-Blue IFN-α/β™ reporter cell assay, and TNFα cytokines were detected by ELISA. The results are reported as the mean ± SD (n = 4 independent experiments).

To directly address the role of BCAP in the regulation of IFN-I production induced by LPS, we moved to a murine immortalized myeloid cell progenitor (Hoxb8) [6] to knock out BCAP expression. Following a procedure previously established in our group [7], Hoxb8 precursors were transduced with a lentiviral vector encoding CAS9 and sgRNA guides specific to BCAP. Following selection, we identified two clones that had lost BCAP expression (Figure 5A). Progenitors (wild type (WT) or BCAP^−/−^) were differentiated into macrophages and stimulated with LPS alone or graded doses of cGAMP. We failed to detect IFN-I production in cells stimulated with LPS or cGAMP alone. Conversely, pre-exposure to cGAMP induced a dose-dependent production of IFN in response to LPS, in line with our previous data [5]. Notably, BCAP-null cells tended to produce less IFN following LPS stimulation at every tested dose. We conclude that BCAP acts as a positive regulator of IFN production upon engagement of TLR4 in cells that underwent prior activation of IFN signaling (Figure 5B). We next evaluated the production of other inflammatory cytokines using the same experimental design. WT and BCAP^−/−^ macrophages were stimulated with either LPS or cGAMP alone or with LPS following overnight pre-exposure to graded doses of cGAMP. In contrast to IFN, the level of TNFα was greatly higher in BCAP^−/−^ cells at all doses tested (Figure 5C). Although not significant, the overall trend is discernible from the presented graph and aligns with the biological effect described. This result indicates that BCAP has a complex role in regulating cellular activation downstream of LPS, promoting IFN at the expense of inflammatory cytokines.

## 3. Discussion

Through ex vivo experiments in patients with SLE and in cultured cells induced to produce IFN-I, we have shown that BCAP is a component of ISGs. Indeed, the inhibition of signaling downstream of IFN-I, both with IFNAR-blocking antibodies and with a JAK inhibitor, prevents an increase in BCAP expression after stimulation of the cGAS-STING-TBK1 pathway, proving that BCAP is an ISG.

In autoimmune diseases, ISGs have drawn interest for their ability to define a transcriptome signature that is used to indirectly evaluate the degree of IFN-mediated inflammation in conditions such as Sjogren’s disease, dermatomyositis, and SLE. Nevertheless, in physiological contexts, ISGs play a role in the innate immune response against intracellular microorganisms, especially viruses. Examples of ISGs are proteins that disrupt viral replication inside the cell, alter membrane characteristics to decrease virus entry and diffusion, and stimulate the activation of lymphocytes and macrophages [8].

Yet the function of BCAP in this context has not been clarified. Our earlier findings showed that DNase2 deficient fibroblasts exhibit a significant increase in IFN production when stimulated with LPS, contrary to the typical response observed in healthy controls’ fibroblasts, which prevalently upregulate TNFα. We hypothesized that in DNase2-deficient cells this behavior might be attributed to the background activation of the cGAS-STING-TBK1 pathway due to the leakage of undigested DNA from lysosomes. Indeed, significant production of IFN was measured in wild-type fibroblasts that were stimulated with LPS following prior incubation with cGAMP, thereby mimicking the cGAS-STING-TBK1 background activation found in DNase2-deficient cells. Our findings indicate that BCAP was significantly upregulated in both DNase2 fibroblasts and in fibroblasts exposed to cGAMP. Accordingly, we hypothesized that this overexpression might be associated with the heightened IFN response to LPS [5]. This notion is consistent with prior evidence indicating that BCAP can redirect cytokine synthesis toward IFN-I [4].

To further clarify this aspect, we generated BCAP^−/−^ HoxB8-derived macrophages in order to investigate the role of the protein in the increased IFN response to LPS observed in cGAMP-primed cells. We demonstrated that LPS stimulation of cGAMP-primed BCAP^−/−^ cells results in a significantly lower increase in IFN-I production compared with wild-type cells. In contrast, TNFα secretion in these settings tended to be higher in BCAP^−/−^ cells. This indirectly establishes the function of BCAP in directing the cytokine pattern generated by LPS toward IFN rather than TNFα.

The mechanism by which increased expression of BCAP can influence the cytokine response pattern is not fully understood. Various pieces of evidence show that BCAP belongs to the superfamily of adaptors containing a TIR domain, involved in TLR signaling [1,2]. BCAP is capable of mediating the activation of the PI3K/AKT pathway after stimulation with Toll-like receptor ligands, leading to reduced production of TNFα and other inflammatory cytokines [2]. After TLR stimulation, the phosphorylation of BCAP is critical for the recruitment and activation of PI3K [9]. In this way, BCAP negatively regulates the maturation of dendritic cells and the antibacterial cytokine response [10]. On the contrary, BCAP-deficient macrophages show increased production of cytokines, such as IL-6, IL-12, and TNFα, after stimulation with LPS [2]. Similarly, a defect in PI3K-AKT-mTOR signaling can also lead to a hyperinflammatory response, likely due to reduced phosphorylation of AKT and, consequently, the transcription factor FoxO1 [11]. Accordingly, the knockdown of FoxO1 in dendritic cells is also associated with reduced pro-inflammatory activity.

Notably, the overexpression of BCAP is linked to a change in cytokine production toward IFN in response to TLR4 stimulation, in addition to the reduced production of pro-inflammatory cytokines [4].

Our results establish a link between the increased IFN response and the reduced pro-inflammatory response related to the action of BCAP. Furthermore, they suggest, for the first time, the idea that BCAP can be considered a key molecule at the center of a positive feedback mechanism that could be implemented, for example, by cells with intracellular bacterial infections, which activate both the cGAS and Toll-like pathways. 

Our findings are consistent with the understanding that inflammatory profiles are inherently complex, often reflecting distinct patterns of infection [12]. It is well-established, for instance, that the release of danger-associated molecular patterns like ATP can significantly modulate a cell’s response to co-occurring bacterial molecular patterns [13]. Therefore, it is not surprising that the activation of intracellular nucleic-acid sensors can influence how a cell responds to extracellular LPS.

While these intricate mechanisms are crucial for combating a vast array of infections, their dysregulation can have detrimental effects in pathological conditions. Consider certain rheumatological diseases where, even without an active intracellular infection, there is an abnormal cytoplasmic stimulation by endogenous nucleic acids, often due to increased release or impaired clearance. In such scenarios, an extracellular LPS challenge could inadvertently trigger an exaggerated IFN response. This improper surge of IFN can then exacerbate the underlying inflammatory diseases, establishing a vicious cycle of inflammation.

These mechanisms can explain the observation of an increased IFN response in cells from patients with SLE or interferonopathies stimulated with LPS [5,14]. Further experiments are necessary to investigate and characterize the synergistic process of the stimulus response from a temporal perspective. These results pave the way for the possibility of studying pharmacological interventions with PI3K-AKT-mTOR pathway inhibitors to modulate the inflammatory response in diseases characterized by IFN hyper-responsiveness. In this regard, recent studies have shown that the inhibition of mTOR through sirolimus is capable of suppressing the hyperproduction of IFN-I in monocytes from subjects with SLE [15]. Considering previous studies on monocyte gene expression modulation in SLE conditions [16], as well as the established efficacy and safety of sirolimus in SLE patients [17], sirolimus-mediated mTOR inhibition could be considered a potential therapeutic strategy in the management of SLE-related inflammation.

## 4. Materials and Methods

### 4.1. Cell Cultures, Stimuli, and Inhibitors

Skin fibroblasts obtained from healthy controls were cultured in RPMI medium with 10% fetal bovine serum (FBS), 100 U/mL penicillin, 100 μg/mL streptomycin, 2 mM L-glutamine (all EuroClone, Milan, Italy), and normocin^®^ (100 μg/mL, InvivoGen, San Diego, CA, USA). 

Cells were seeded at a density of 2 × 10^5^ in complete medium (with 10% low-endotoxin FBS, Microtech, Naples, Italy) and treated with different stimuli. At the end of the incubation periods, fibroblasts were recovered with trypsin-EDTA (EuroClone, Milan, Italy) and centrifuged at 300 *g* for 10 min at room temperature, stained with Trypan Blue (Lonza, Basel, Switzerland), and counted using a disposable slide (Fast Read 102, Biosigma, Venice, Italy).

To verify *PIK3AP1* expression, fibroblasts were treated with scalar concentrations of IFNα2a (10, 100 U/mL, Miltenyi Biotec, Bergisch Gladbach, Germany) for 6 h and 24 h.

To prevent the transcription of IFN-stimulated genes after stimulation of the IFN pathway, fibroblasts were incubated either with 100 nM tofacitinib (Sigma Aldrich, Darmstadt, Germany) at 37 °C for 1 h or with scalar concentrations of the monoclonal antibody anifrolumab (0.001, 0.01, 0.1, 1 ug/mL, Saphnelo, Cambridge, UK). The stimulation of the cGAS-STING-TBK1 pathway was obtained with 50 µg/mL cGAMP (Invivogen, San Diego, CA, USA).

### 4.2. Generation on HoxB8 BCAP Knockout Cells

The sgRNA PIK3AP1 genome editing guides were designed using the Broad Institute website (http://www.broadinstitute.org, accessed on 1 April 2021), and off-target site prediction was cross-checked in Benchling (http://benchling.com, accessed on 1 April 2021). Two different sgRNAs were cloned in the LentiCRISPR-v2 plasmid (#52961, Addgene, Watertown, MA, USA); the sgRNA sequences are reported below (5’ → 3’).

For caccgTTCCCAGGCGATATCTCCGG Rev aaacCCGGAGATATCGCCTGGGAAc Exon 3
For caccgAGAACAACACCATCCCTATG Rev aaacCATAGGGATGGTGTTGTTCTc Exon 7

To generate BCAP knockout (BCAP^−/−^) clones, 3 × 10^5^ HoxB8 cells at the precursor stage were infected with lentiviral particles encoding BCAP-targeting guides. Cells were placed under puromycin selection and single cells cloned to isolate individual clones (10 μg/mL). Ten single clones were expanded and differentiated into macrophages to analyze BCAP expression by WB. Two clones showing BCAP expression, and two clones showing BCAP deletion were retained for further assays and named WT or BCAP^−/−^.

### 4.3. Hoxb8 Differentiation in GM-CSF

To obtain macrophages, WT or BCAP^−/−^ Hoxb8 precursors were cultured at a concentration of 1.5 × 10^6^ cells/mL in non-treated 6-well-plates for 7 days using IMDM medium supplemented with 10% FBS, 50 μM 2-mercaptoethanol, and 1% gentamicin, complemented with 30% of the supernatant of the GM-CSF produced from the J558 cell line. The cells were used for experiments on day 7.

### 4.4. Western Blot Analysis

Cells were washed and lysed with a buffer containing 50 mM Tris-HCl (pH 7.6), 150 mM NaCl, 0.1% SDS, 1% NP-40, and protease/phosphatase inhibitors. The protein concentration was determined by a Bradford Pierce BCA protein assay (Thermo Fisher Scientific, Waltham, MA, USA) according to the manufacturer’s instructions. The supernatants were boiled for 10 min and separated by SDS-PAGE. Polyacrylamide gels were cast with GERBU acrylamide M-Bis 30% solution, and proteins were spread on running gel at 12%. Primary antibodies included αBCAP (sc-515498, Santa Cruz Biotechnology Inc., Dallas, TX, USA) and αGapdh-HRP (#G8795, Sigma Aldrich, Darmstadt, Germany), and as secondary antibody the αMouse-HRP (#A10551, Jackson Laboratory, Bar Harbor, ME, USA).

### 4.5. Functional Assays of Activation in Hoxb8-Derived Macrophages

To induce activation of macrophages, 2 × 10^5^ cells were plated in a U-bottom 96-well plate and primed with a STING agonist (cGAMP, Invivogen, San Diego, CA, USA) at different dilutions (0.025, 0.5, 1, 2 μg/mL) for 24 h in 100 μL at 37 °C. Following the primary stimulation, cells were washed and re-stimulated with 0.1 μg/mL LPS (Invivogen, San Diego, CA, USA) for 24 h at 37 °C.

The cell culture supernatant was harvested to dose the cytokine content. For TNFα detection, a 1/10 dilution was developed using the ELISA Max Standard sets (BioLegend, San Diego, CA, USA), following the manufacturer’s instructions. The absorbance was read at 450 nm with an iMark Microplate Absorbance Reader (Bio-Rad, Hercules, CA, USA). To detect IFN-I, the pure cell culture supernatant was analyzed using B16-Blue IFN-α/β™ reporter cells, according to the manufacturer’s instructions. IFN-β (BioLegend, San Diego, CA, USA) (0–20 ng/mL) was added to generate a standard curve.

### 4.6. Measure of PIK3AP1 Expression by Real-Time PCR

The measurement of the expression of *PIK3AP1* was carried out on RNA obtained from cellular experiments in cultured fibroblasts and from blood samples of patients with SLE. 

For cell cultures, RNA was purified from 2 × 10^5^ cells using the High Pure miRNA Isolation Kit (Roche, Basel, Switzerland) and retro-transcribed (to a maximum of 1 μg of RNA) using the SensiFAST cDNA Synthesis Kit (Bioline, Norwich, UK). The obtained cDNAs were stored at −20 °C. 

Evaluations of *PIK3AP1* expression and IFN score values were also conducted in a collection of 36 cDNA samples obtained from patients with pediatric SLE at the IRCCS Burlo Garofolo (Italy) and at the Ribeirão Hospital Preto (Brazil) during a previous research project by our group (see Table S1 for a summary of the clinical and laboratory data). 

Furthermore, 20 healthy young adult subjects were included as a control group at the IRCCS Burlo Garofolo, using RNA stabilization tubes (PAXgene Blood RNA Tubes, PreAnalytiX, Hombrechtikon, Switzerland). Following purification with the dedicated kit (PAXgene Blood RNA Kit, PreAnalytiX, Hombrechtikon, Switzerland), RNA was quantified by NanoDrop (Thermo Fisher Scientific, Waltham, MA, USA) and retro-transcribed (up to 1 μg of RNA total) using the SensiFAST cDNA Synthesis Kit (Bioline, Norwich, UK). The obtained cDNA samples were stored at −20 °C. *PIK3AP1* gene expression, as well as *ISG15* as a positive control for ISG evaluation, was assessed in cDNA samples by real-time PCR (qPCR) using the CFX Opus 96 instrument (BioRad, Hercules, CA, USA), TaqMan Gene Expression Master Mix (Thermo Fisher Scientific, Waltham, MA, USA), and TaqMan probes (Thermo Fisher Scientific, Waltham, MA, USA). Using Maestro software Version 2.3 (Bio-Rad, Hercules, CA, USA), the quantities of target genes were normalized with the expression levels of two housekeeping genes, *HPRT1* and *G6PD*. A relative quantitative analysis of the gene expression was conducted using the 2^−∆∆Ct^ method [18].

### 4.7. Calculation of the IFN Score by Real-Time PCR

An IFN signature assessment was performed by analyzing the expression of 6 ISGs (*IFI27*, *IFI44L*, *IFIT1*, *ISG15*, *RSAD2*, and *SIGLEC1*) by qPCR with the same instruments and reagents described in the previous paragraph (Section 4.6). The amounts of target genes were normalized with the expression levels of two housekeeping genes, *HPRT1* and *G6PD*. A relative quantitative analysis of the gene expression was conducted using the 2^−∆∆Ct^ method compared with the control group. The IFN score, corresponding to the median of the relative quantifications of the 6 genes analyzed, was calculated for each subject, allowing the level of IFN inflammation to be considered as positive or negative in relation to a normal reference range calculated from healthy controls (positive score > 2.2) [19].

### 4.8. Statistics

Data analysis was performed using GraphPad Prism 8 software. *p*-Values < 0.05 were considered as statistically significant.

## 5. Conclusions

Our findings indicate that BCAP is a gene that is activated by IFN and is implicated in a potential amplification loop of inflammation triggered by IFN in the context of bacterial infections. Indeed, the upregulation of BCAP is linked to a change in the cytokine secretion following LPS stimulation, characterized by heightened IFN synthesis and decreased TNFα synthesis. Physiologically, this action may enable the cell to enhance IFN response when an intracellular infection, which activates cGAS, is concomitant with the presence of bacterial material outside the cell, activating TLR4. In pathological conditions, such as SLE, this feature may explain the intensification of IFN-mediated inflammation after bacterial infections, which appears to be a major contributor to the resurgence of disease activity. 

## Data Availability

Data will be provided from the corresponding author upon reasonable request.

## Supplementary Materials

The supporting information can be downloaded at: https://www.mdpi.com/article/10.3390/ijms26157034/s1.

## Abbreviations

The following abbreviations are used in this manuscript:

BCAP	B-Cell Adapter for PI3K
cGAMP	Cyclic GMP-AMP
cGAS	Cyclic GMP-AMP Synthase
CI	Confidence Interval
IFN-I	Type I Interferon
IL	Interleukin
ISGs	Interferon-Stimulated Genes
LPS	Lipopolysaccharide
mTOR	Mammalian Target of Rapamycin
PI3K	Phosphatidylinositol 3-Kinase
PIK3AP1	PI3K Adapter Protein 1
TBK1	TANK-Binding Kinase 1
TIR	Toll-IL1-receptor
TIRAP	TIR adaptor protein
TLRs	Toll-Like Receptors
TNFα	Tumor Necrosis Factor α
SLE	Systemic Lupus Erythematosus
STING	Stimulator of Interferon Genes
