# Supplementary material for: BCAP Is an Interferon-Stimulated Gene That Enhances Type I Interferon Activity in Response to Lipopolysaccharide
Source: Int J Mol Sci. 2025 Jul 22;26(15):7034. doi: 10.3390/ijms26157034 (PMC12346107; doi:10.3390/ijms26157034)

Supplementary Table S1. Relevant data of 36 subjects with Systemic Lupus Erythematosus (SLE).

Patient cod.	SLEDAI	anti dsDNA autoantibodies	Hypo-complementemia	C3 (0.9-1.8 g/L)	C4 (0.1-0.4 g/L)	Steroids	Other treatments
SLE_01	29	1	1	0.43	0.07	1	AZA, MMF, RIX, TAC
SLE_02	8	1	0	1.26	0.30	0	HCQ
SLE_03	8	0	0	1.41	0.24	1	AZA, HCQ
SLE_04	0	0	0	1.44	0.14	1	HCQ, MMF
SLE_05	0	0	0	1.21	0.23	1	HCQ, MMF
SLE_06	2	0	1	1.13	0.01	0	HCQ
SLE_07	0	0	0	1.10	0.16	0	HCQ
SLE_08	2	0	1	0.88	0.13	1	AZA, HCQ, MMF
SLE_09	4	1	1	0.74	0.07	1	AZA, HCQ, MMF
SLE_10	4	0	0	1.08	0.14	1	HCQ, MMF
SLE_11	9	1	1	0.67	0.12	1	AZA, HCQ, MMF
SLE_12	0	0	0	1.35	0.30	1	AZA, HCQ
SLE_13	10	1	1	0.65	0.06	1	AZA, HCQ
SLE_14	2	1	0	1.29	0.20	1	HCQ
SLE_15	10	1	1	0.79	0.07	1	AZA, HCQ
SLE_16	0	0	0	1.22	0.14	0	HCQ
SLE_17	32	1	1	0.36	0.04	1	HCQ
SLE_18	4	1	0	0.97	0.13	1	HCQ
SLE_19	6	1	0	1.19	0.11	1	HCQ
SLE_20	10	1	0	0.91	0.13	1	HCQ, MMF
SLE_21	16	1	1	0.43	0.04	1	HCQ, MMF, RIX
SLE_22	8	1	1	0.85	0.22	1	AZA, HCQ, MMF, TAC
SLE_23	2	1	0	1.10	0.16	1	HCQ, MTX
SLE_24	5	0	0	0.96	0.14	0	HCQ, MMF
SLE_25	6	1	0	1.22	0.27	1	AZA, HCQ
SLE_26	2	1	0	1.51	0.21	1	AZA, HCQ
SLE_27	2	0	0	0.98	0.21	1	AZA, HCQ
SLE_28	22	1	1	0.72	0.08	1	AZA, HCQ, MMF, RIX, TAC
SLE_29	10	1	0	1.50	0.27	1	HCQ
SLE_30	4	0	0	1.12	0.25	1	HCQ, MMF, RIX
SLE_31	16	1	1	0.36	0.09	1	HCQ
SLE_32	4	1	1	0.53	0.30	1	MMF
SLE_33	2	0	1	1.36	0.10	1	HCQ, MMF
SLE_34	15	1	1	0.25	0.05	1	HCQ, MMF, RIX
SLE_35	14	1	0	1.10	0.65	1	MMF
SLE_36	8	1	1	0.67	0.60	1	HCQ, MMF

1 = present; 0 = not present.

SLEDAI: Systemic Lupus Erythematosus Disease Activity Index; AZA: Azathioprine; HCQ: Hydroxychloroquine; MTX: Methotrexate; MMF: Mycophenolate Mofetil; RIX: Rituximab; TAC: Tacrolimus.

Supplementary Figure S1. BCAP protein expression in HoxB8 transduced cells after differentiation in macrophages.
Blot shows all clones after BCAP infection compared to WT as a control. The black square indicates the clones shown in Figure 5, including the two selected as BCAP$^{-/-}$.

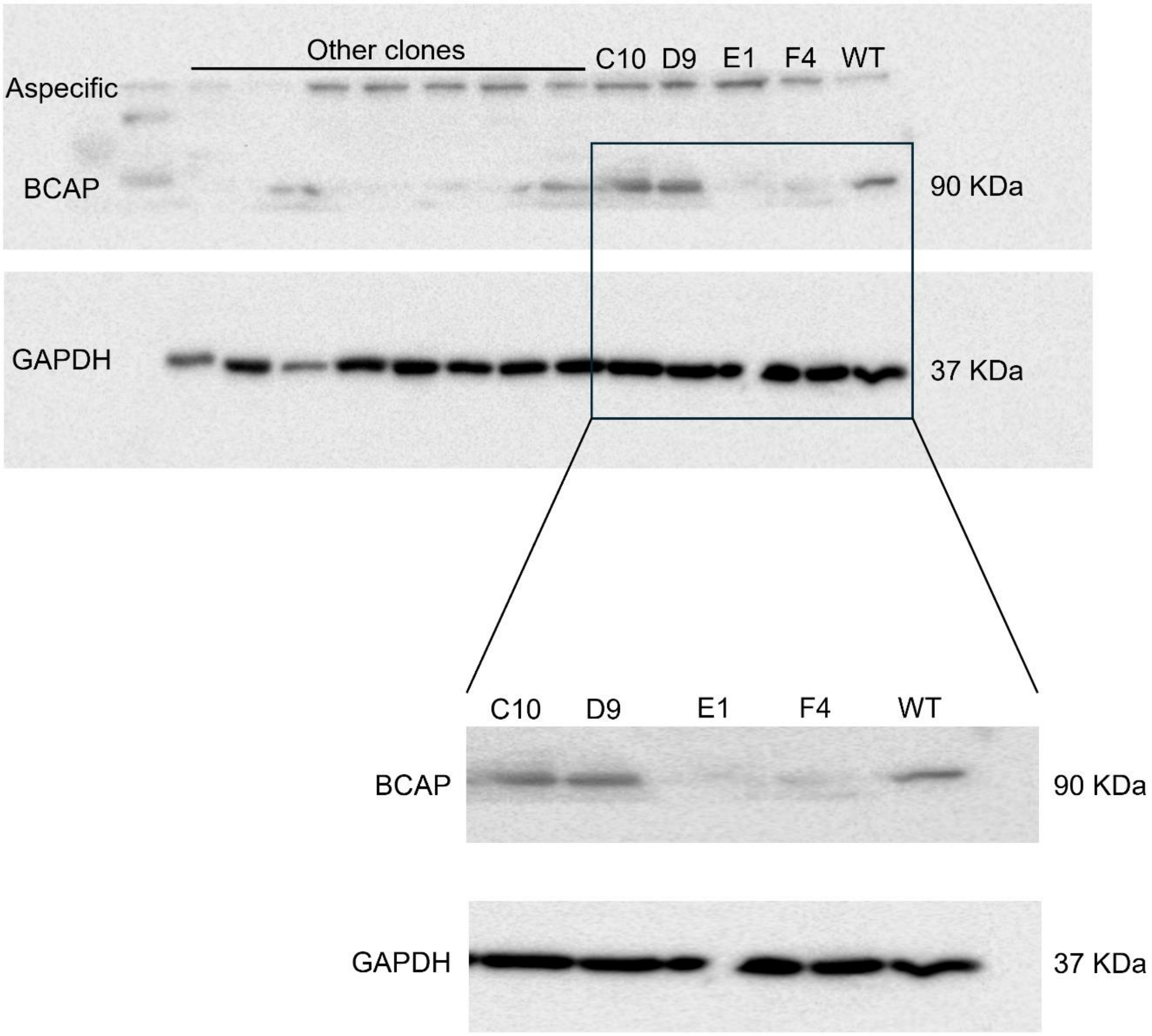

Supplement: Supplementary file 1 [file ijms-26-07034-s001.zip › ijms-3745725-supplementary.pdf]
